# Monostotic fibrous dysplasia of jaw bones: a case series

**DOI:** 10.1186/s12903-024-04894-3

**Published:** 2024-09-19

**Authors:** Alka Hande, Padmashri Kalmegh, Swati Patil, Archana Sonone, Aayushi Pakhale

**Affiliations:** Department of Oral & Maxillofacial Pathology and Microbiology, Sharad Pawar Dental College & Hospital, Datta Meghe Institute of Higher Education and Research, Sawangi (Meghe), Wardha, 442004 Maharashtra India

**Keywords:** Fibro-osseous lesions, Fibrous dysplasia, GNAS1, Monostotic, Polyostotic, Malignant change

## Abstract

**Background:**

Fibrous dysplasia (FD) is a benign fibro-osseous lesion, a skeletal developmental anomaly of the bone-forming mesenchyme. The diagnosis of fibro-osseous lesions, particularly those of the jaw bones, poses significant challenges to clinicians and pathologists since it requires a correlation of clinical, radiological, histological, and surgical findings. Accurate and specific diagnosis is crucial as treatment modalities differ with different fibro-osseous lesions.

**Methods:**

This retrospective analysis presents a case series of a rare condition of monostotic FD in the maxillofacial region affecting jaw bones diagnosed and/or treated over period of 10 years.

**Results:**

Five cases of monostotic FD were diagnosed and treated between a period of 2013 and 2023. The cases from the 2nd to 8th decade were included in the analysis with equal involvement of males and females. Out of five cases, four cases were involving maxilla and 1 showed involvement of mandible.

**Conclusion:**

FD is a rare entity affecting the jaw bones which often lead to disfigurement of face. Early detection is warranted to decrease potential complications. In addition, genetic analysis could help in understanding the occurrence in certain population.

## Highlights

Fibrous dysplasia presents clinically in varied forms which may appear as the collision of different pathologic processes.

We present a case series of a rare condition of monostotic fibrous dysplasia in the maxillofacial region affecting jaw bones- over the period of 10 years of an institutional database.

We discussed the present knowledge on fibrous dysplasia, emphasizing the need to understand its clinical, radiographic, and histological characteristics, as well as essential aspects relevant to differential diagnosis and patient management.

## Introduction

The term “benign fibro-osseous lesions” (BFOLs) refers to a wide variety of pathologic disorders that include developmental lesions, reactive or dysplastic diseases, and neoplasms [1]. Fibrous dysplasia (FD), cemento-osseous dysplasia, and ossifying fibroma are some of the most prevalent fibro-osseous lesions [2]. Regardless of subtype, all BFOLs indicate fibrous connective tissue replacement of normal bone with an admixture of mineralized tissue, including osteoid, mature bone, and/or cementum-like calcifications [3]. FD is a hamartomatous, non-neoplastic developmental defect of unknown origin in which normal bone is replaced by an aberrant proliferation of cellular fibrous connective tissue intermixed with varying bony trabeculae [4, 5]. Von Recklinghausen first discovered “osteitis fibrosa generalisata” in 1891 in a patient with skeletal abnormalities caused by fibrotic bone alterations. In 1938, Lichtenstein first used the term “fibrous dysplasia” to describe the condition [6]. Fibro-osseous dysplasia and fibrous osteodysplasia are two terms that can be used interchangeably to describe FD [7]. Reed characterises the condition as “arrest of bone maturation in woven bone with ossification caused by nonspecific fibro-osseous metaplasia” [8]. Schlumberger defined the disease process as “monostotic fibrous dysplasia” when it originally involved a single bone [9]. FD accounts for approximately 2.5% of all bone lesions and 7% of benign bone tumors, with a prevalence of 1:4000-1:10,000 incidence [10]. This rare benign skeletal condition shows involvement of one or more bones that is monostotic or polyostotic respectively. Polyostic form is further classified into 3 subtypes i) “ Multiple-bone Jaffe-Lichtenstein ”, which is sometimes associated with “café au lait” pigmentation (coffee with milk) (ii) “McCune-Albright’s syndrome” is distinguished by widespread involvement of skeleton, café au lait pigmentation, and a number of endocrinopathies, the most frequent precocious puberty (iii) Mazabraud syndrome which is characterized by polyostotic FD and intramuscular myxomas. During the phase of growth and maturation of body mostly FD remains dormant and there is burnout phase during early adulthood (late adolescence or early twenties), corresponds with growth milestones [11]. Overproduction of the enzyme tyrosinase, which is the rate-limiting step in melanin production, results in café-au-lait spots [12]. The ratio of polyostotic to monostotic forms varies between 8:1 and 10:1 respectively [13]. Facial and cranial bones are involved in about 50% of patients with polyostotic FD and 10–27% of patients with monostotic FD [14]. In the monostotic FD the femur, tibia, ribs, calvaria, skull base, and in orofacial bones, zygoma, maxilla and mandible are commonly affected. Amongst the jaw bones in 50% of patients, the maxilla is more commonly affected than the mandible [15, 16]. The majority of these lesions are unilateral affecting the posterior region [7]. In addition to monostotic and polyostotic FD, “craniofacial FD” is a presentation in which the lesions are confined to adjacent bones of the craniofacial skeleton [17]. Craniofacial FD is not totally monostotic due to the involvement of several contiguous craniofacial skeleton bones, but it is also not truly polyostotic because bones other than the craniofacial complex are not frequently involved [18]. Both endochondral and desmal ossifying bones can develop FD. The most common symptom of craniofacial FD is prominent swelling in the affected area [19]. Its onset is frequently associated with rapid skeletal growth and exhibits a self-limiting pattern, because once bone growth ceases, FD becomes more quiescent or static. The most typical presenting characteristic of monostotic FD is painless expansion in the affected region, however, it can occasionally progress rapidly [20]. The monostotic type affects both sexes equally, but the polyostotic variant has a female predilection [21]. Because of the oestrogen receptors identified in FD, female patients have elevated pain levels during pregnancy and the menstrual cycle [22].

## Methods

**Fig. 1:**
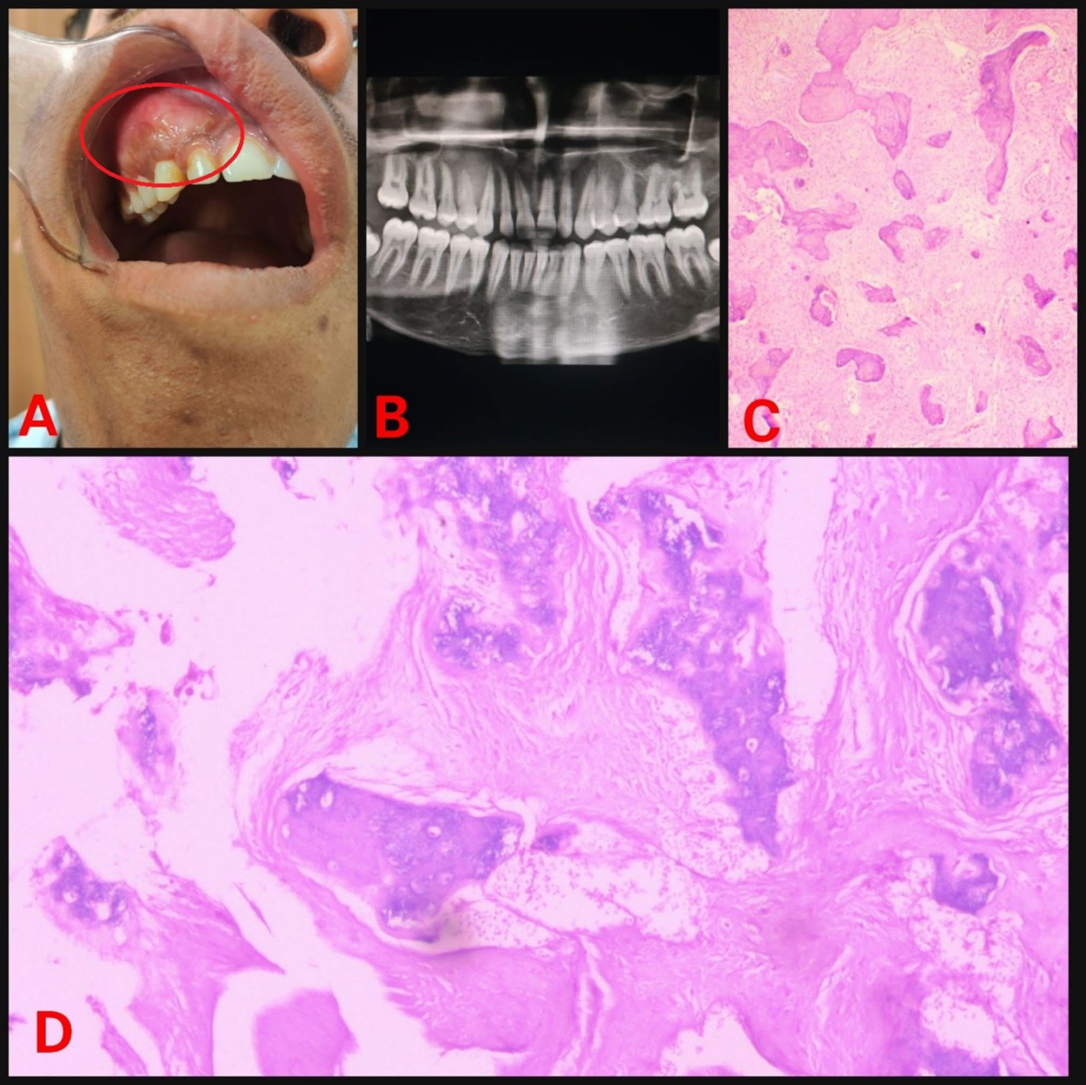
(**A**) Intraoral swelling (**B**) Orthopantomogram (OPG) showing radioopaque lesion (**C**) Hematoxylin and Eosin (H & E) staining under low power view (**D**) H & E staining under high power view

**Fig. 2:**
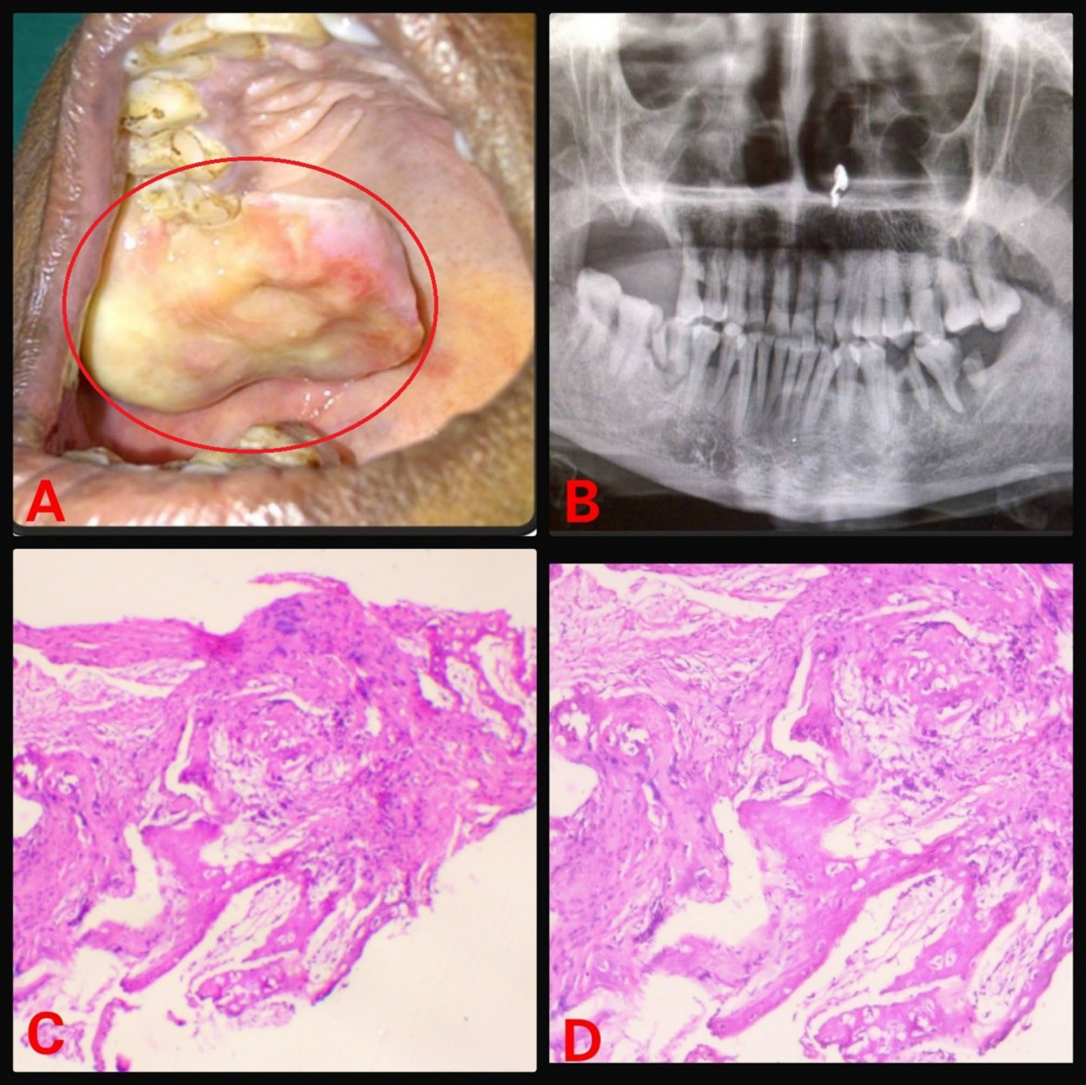
(**A**) Intraoral swelling (**B**) Orthopantomogram (OPG) showing radiolucent lesion (**C**) Hematoxylin and Eosin (H & E) staining under low power view (**D**) H & E staining under high power view

**Fig. 3:**
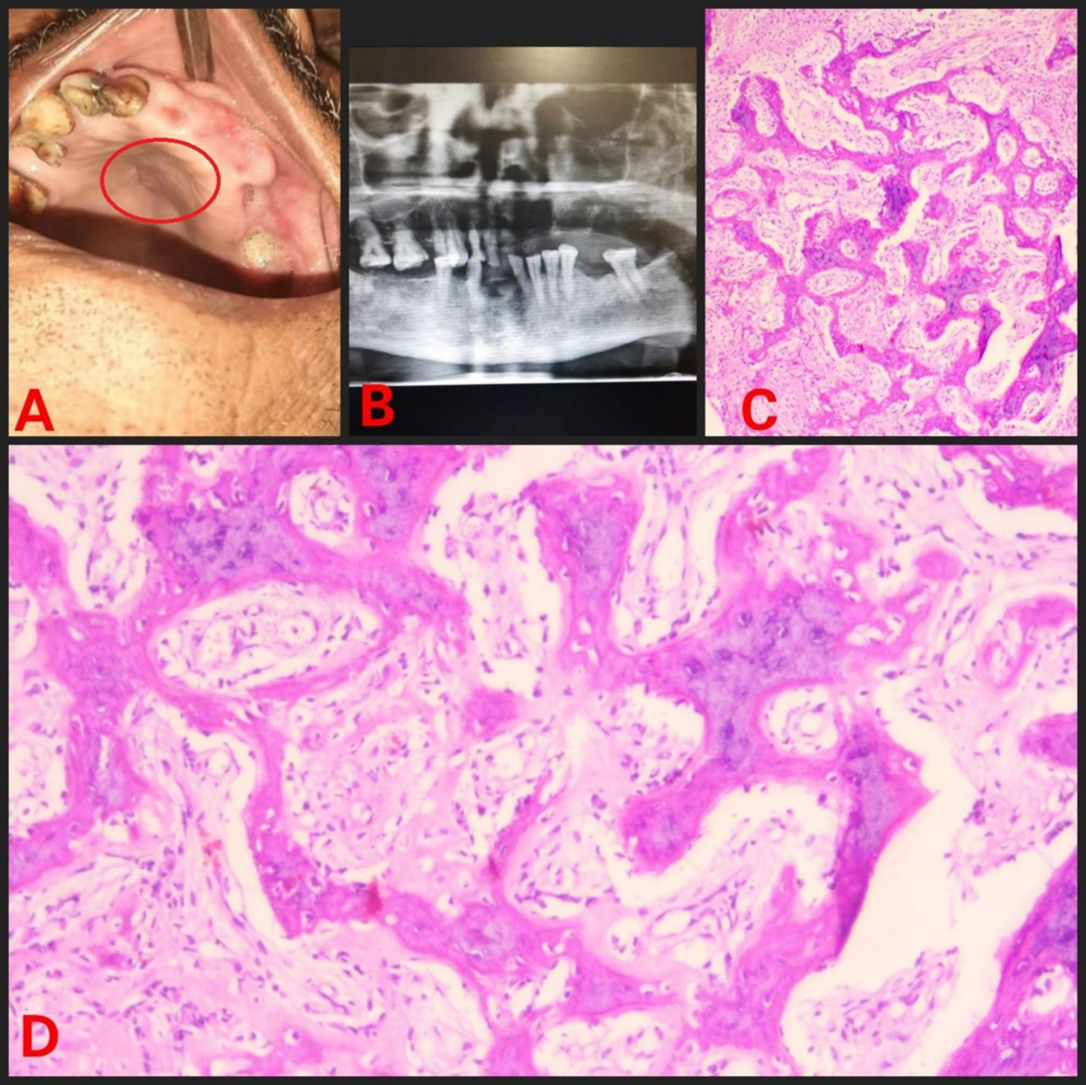
(**A**) Intraoral swelling (**B**) Orthopantomogram (OPG) showing radiolucent lesion (**C**) Hematoxylin and Eosin (H & E) staining under low power view (**D**) H & E staining under high power view

**Fig. 4:**
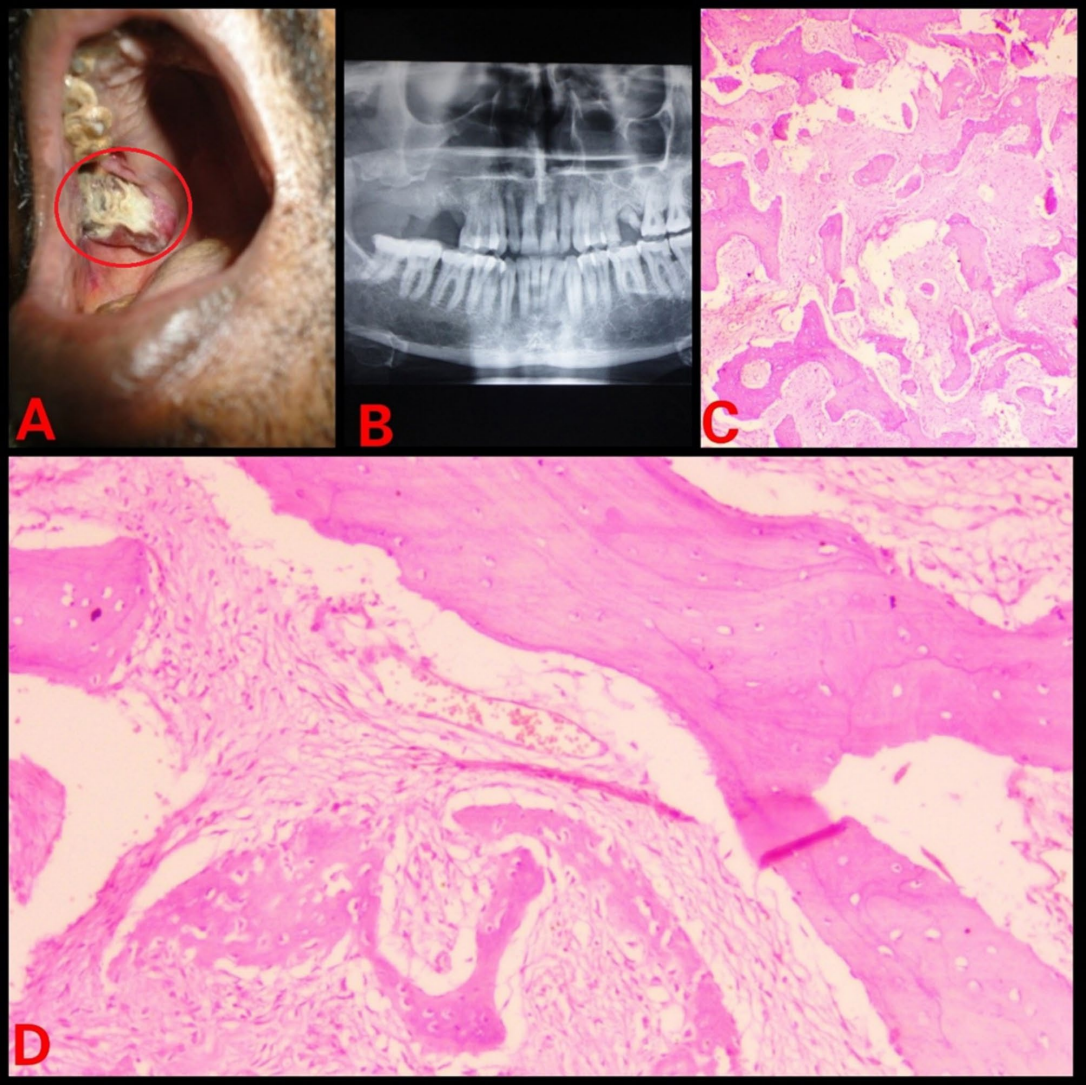
(**A**) Intraoral swelling (**B**) Orthopantomogram (OPG)  showing radiolucent lesion with areas of radiopacity  (**C**) Hematoxylin and Eosin (H & E) staining under low power view (**D**) H & E staining under high power view

**Fig. 5:**
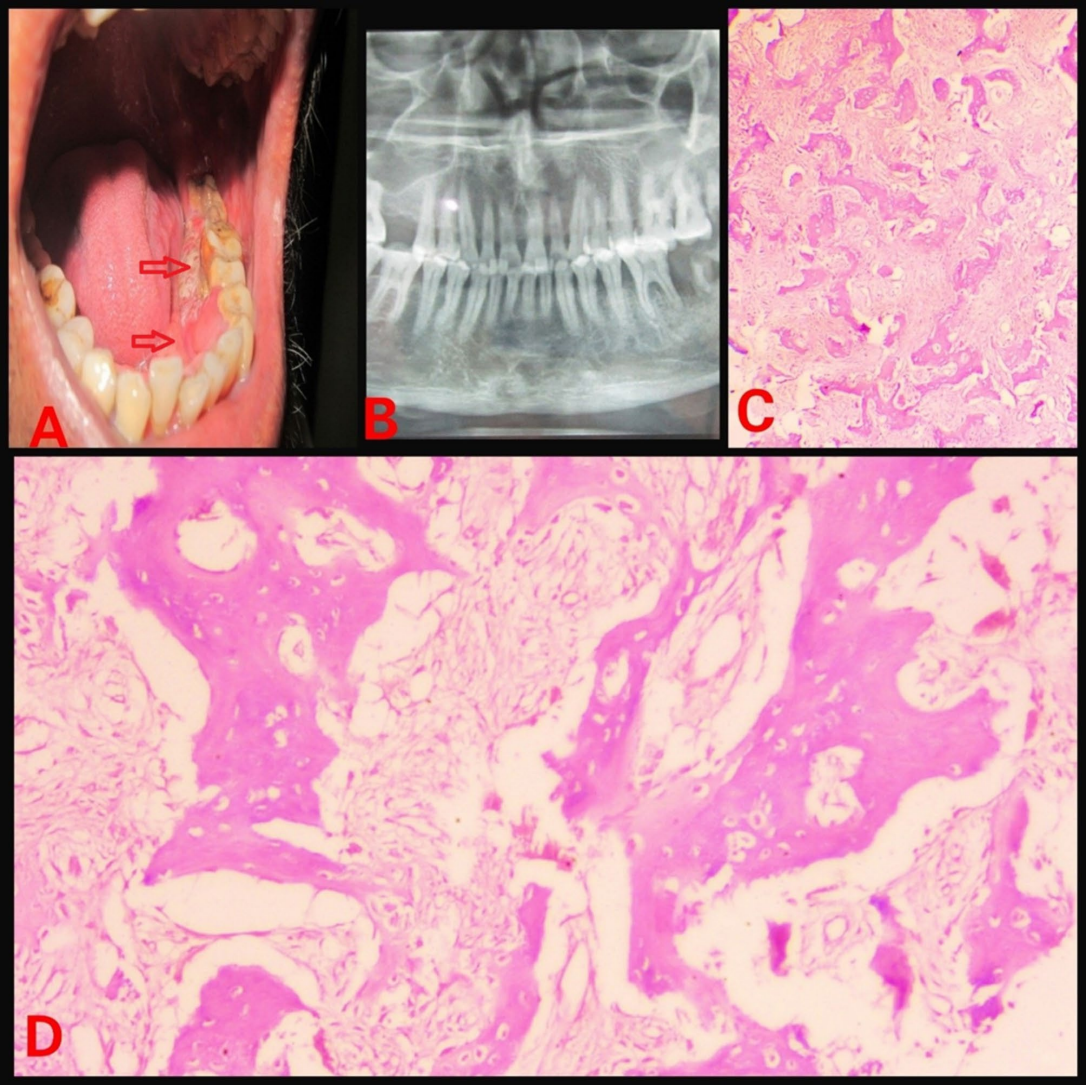
(**A**) Intraoral swelling (**B**) Orthopantomogram (OPG)  showing radiolucent lesion (**C**) Hematoxylin and Eosin (H & E) staining under low power view (**D**) H & E staining under high power view

The retrospective analysis of institutional medical records from 2013 to 2023 were reviewed for FD cases. Ethical approval was obtained from the IEC. Demographic details, medical, dental history of each case were retrieved from the database. Further, radiographic and histopathology features of case were reviewed again to rule out any other diagnosis. Treatment, disease progression and follow-up information were also gathered. In 2023, a 16-year-old male patient reported with painless swelling on the right side of maxillary bone in the posterior aspect that had been present for 3 years. History of the present illness reveals that 3 years ago, the patient first observed small growth over the right maxillary alveolar region. The swelling gradually grew in present size over the course of 3 years. Extraorally, the face was clinically asymmetrical due to swelling on the affected right side. Intraorally, the right side of the alveolus had enlarged, measuring roughly 4 × 2 cm in size (Fig. 1). The enlargement was firm and non-tender on palpation. No historyof trauma or tooth loosening was there. There was no swelling anywhere on the body, and there were no café au lait spots either. The investigations include a complete blood count, blood calcium, serum total alkaline phosphatase (ALP), and a panoramic radiograph. All the parameters, with the exception of ALP, were within normal ranges. ALP levels have risen to 300 U/L. An incisional biopsy of the maxillary lesion had been carried out and processed for histopathological examination. A macroscopic examination of biopsy tissue revealed a single piece of firm tissue that was creamish white in color and size of 1 × 0.8 × 0.4 cm. Depending on the clinical history, radiographic evaluation, and histological features of the lesion the diagnosis of monostotic fibrous dysplasia (FD) was made. Post diagnosis the patient was posted for surgical management. Further a retrospective analysis of 5 cases diagnosed with monostotic FD in a tertiary healthcare center was conducted, with clinical findings, radiological characteristics, histological features, and biochemical analysis like serum alkaline phosphatase. All pertinent data from each patient was collected and tabulated (Table 1). Among the 5 cases, the disease was restricted to the maxilla in 4 cases (Figs. 1, 2, 3 and 4), whereas 1 case showed mandibular involvement (Fig. 5). There was no history of pain in any of the cases. All the included cases were from the 2nd to 8th decade of life. In context to gender predilection, females and males were equally affected in the ratio of 1:1. The age range of all cases were between 16 and 80 years. The broad age range among people may simply indicate the duration at which the lesions were first noticed and identified, rather than a later age of onset of growth. Because most cases of FD are gradual in the beginning, painless, and asymptomatic throughout delayed reporting by the patient which results in a late diagnosis of the lesion. The level of ALP in all patients was significantly greater than the normal range (Normal range – 38–126 U/L). To the best of our knowledge, this report may be the first case series that provides comprehensive, epidemiological, and clinicopathological insight into craniomaxillofacial FD in Central India, which will significantly contribute to the literature on this rare disorder.

**Table 1:** Patient details

Sr. No.	Age/ Sex	Area of chief complaint	Size	Radiographic features	Histopathologic features	Alkaline phosphatase level (Normal range – 38–126 U/L)	Treatment modalities
Patient 1 (Fig. 1)	16/M	Upper right anterior alveolus	4 × 2 cm	Radio-opaque lesion	• Lesion in the mature phase • Curvilinear trabeculae • Osteoblastic rimming is absent	300 U/L	Surgical excision
Patient 2 (Fig. 2)	50/F	Upper right posterior alveolus	4.5 × 4 cm	Radiolucent lesion	• A lesion in the immature phase • Osteocytes are large and collagen fibers of trabeculae are seen extending out into fibrous tissue • Wide osteoids seen in some places • Connective tissue stroma composed of blood vessels and fibroblasts • Osteoblastic rimming is absent	305 U/L	Radical excision and reconstruction
Patient 3 (Fig. 3)	80/M	Mid palatine region	1.8 × 1.2 cm	Radiolucent lesion	• A lesion in the immature phase • Increased number of osteocytes • Open faced active stellate-shaped osteoblasts scattered in fibrous tissue suggest functioning in new bone formation • Osteoblastic rimming is absent	196 U/L	Under treatment with bisphosphonate therapy and follow up
Patient 4 (Fig. 4)	50/M	Upper right posterior alveolus	1.5 × 1 cm	A radiolucent lesion with areas of radiopacity	• Lesion in mixed-phase • Reduced number of osteocytes • Osteoblastic rimming is absent	162 U/L	Under treatment with bisphosphonate therapy and follow up
Patient 5 (Fig. 5)	44/M	Lower left alveolus	3 × 2 cm	Radiolucent lesion	• A lesion in the immature phase • More number of osteocytes • Bony trabeculae of coarse and woven bone Osteoblastic rimming is absent	417 U/L	Conservative bone shaving

## Discussion

The cause of FD is unknown; however, it is anticipated to be a genetic predisposition [7]. The postnatal development of FD does not reflect the embryonic phase during which the genetic change occurred. But it depicts the period when the dynamic balance of genetically altered and normal osteogenic cells occurred [23]. Based on the period of time and pattern of the pathogenic mutations, as well as the type of pluripotent cells that mutate during early embryonic development, FD is believed to be an anomaly of pluripotent embryonic cells [24]. All manifestations of the disease have been linked to two mutations. The mutations in codon 201 replace the arginine residue with cysteine, histidine, or, other amino acids in rare cases. The disease-causing mutations occur postzygotically. This explains why the disease is never inherited and why affected individuals exhibit somatic mosaics [25, 26]. The mutation is assumed to be postzygotic, episodic, and located on the GNAS1 gene (Guanine Nucleotide Binding Protein, Alpha Stimulating). This gene on “chromosome 20q13” is responsible for the production of the “alpha subunit of stimulating G-proteins”. This mutation activates adenylate cyclase, which raises intracellular cyclic adenosine monophosphate (AMP) levels, leading to aberrant osteoblast differentiation and the development of metaplastic bone [7]. When the GS gene (GNAS1) is mutated, it is linked to the FD disease spectrum [27]. The increase in stimulatory G protein activity in osteoblast progenitor cells has been proposed to be the outcome of increased proliferation and aberrant differentiation [28]. The mutations in GNAS1 were not observed in cementoosseous dysplasia, ossifying fibromas, or normal bone, indicating that the mutation is specific to FD [20].

Furthermore, studies have indicated the role of the WNT/-catenin signaling pathway in FD [12]. Bone markers are used to evaluate disease activity and response to therapy. The typical markers in FD include ALP and hydroxyproline (OHP). ALP is reported to be elevated in approximately 75% of patients, with the level being linked with severity and activity of the disease [29]. The concentration of ALP may be a significant marker for detecting the recurrence of FD in an early stage. ALP levels are much greater in individuals with craniofacial FD and may be an effective marker to assess cancer progression in it [30]. Patients with FD may also have higher levels of osteocalcin (OC), deoxypyridinoline (D-Pyr), and the C-telopeptide of type I collagen breakdown products (CTX) [31]. The bone matrix in FD is deficient in osteopontin and bone sialoprotein (BSP) as compared to normal bone. BSP is an osteoblastic cell differentiation marker that is necessary for mineralization [29, 32]. The FD-associated phosphate degeneration results from abnormal osteogenic precursors overproducing Fibroblast growth factor-23 (FGF23), which is secreted by both osteoblasts and osteocytes in normal bone [33].

### Histopathological spectrum 

Microscopically, fibro-osseous lesions are distinguished by the occurrence of varied dense collagenous tissue adjacent to osseous trabeculae; nevertheless, the stage of development of the FD has a considerable impact on the microscopic presentation. As aforementioned, a more immature and radiolucent lesion has an increased proportion of fibrous tissue, whereas as the lesion becomes more mature and radiopaque, the proportion of fibrous tissue is decreased and osseous trabeculae dominates [34]. FD is regarded to be an impeded maturation at the woven bone stage [35]. There are three primary histologic patterns of FD: the classic “ Chinese letter ”, the “ pagetoid type ”, and the “ hypercellular ” type [36, 37]. As per literature review these various microscopic features are site-specific, or the variations may be due to mechanical stress. The discrepancies among these histologic types are beyond the scope of this article and have no bearing on patient prognosis [38]. Microscopic identification of fibro-osseous conditions is difficult because of the overlapping histological characteristics of ossifying fibroma, osseous dysplasia, and FD. Therefore FD should be differentiated from simple bone cysts, ossifying fibromas, adamantinoma, low-grade intramedullary osteosarcoma, and Paget’s disease. The simultaneous assessment of clinical, radiographic, and microscopic findings is the gold standard for diagnosing fibro-osseous lesions [20].

The modalities used in the examination of FD include conventional radiography, computed tomography (CT), magnetic resonance imaging (MRI), and bone scintigraphy [39]. The radiographic characteristics of FD vary depending on the ratio of fibrous tissue to mineralized bone in the lesion [40]. Early FD can be unilocular or multilocular radiolucency, and have borders that are either poorly or clearly delineated. FD contains mottled radiopaque patterns with ill-defined borders that blend into normal surrounding bone [23].

Spontaneous malignant transformation of FD is extremely rare, with incidences as low as 0.93% which includes mesenchymal malignancies like osteosarcomas, fibrosarcomas, and chondrosarcomas [20]. Patients with polyostotic FD are more likely to have malignant transformation than patients with monostotic FD [13]. There is currently no treatment available to prevent disease progression or malignant transformation [12].

The clinical history, demographic data, radiological, and histopathologic findings are the important criteria used to make the diagnosis of FD [29]. In case of the challenges for lesional biopsy due to the location of involvement, age, or other considerations (particularly in polyostotic FD or McCune-Albright’s syndrome), radiographic and clinical findings may be required to confirm the diagnosis [39]. Immunohistochemistry is used in the diagnosis of FD to rule out the possibility of a malignant lesion with a significant history. Polymerase chain reaction and other genetic amplification techniques can now be utilized for screening for gene mutations such as GNAS mutation which is beneficial for confirming a diagnosis [22, 41].

The only treatment for FD is to maintain the optimal density of bones by nutrition, physical activity, and therapeutic pharmaceuticals [12]. The recommended treatment includes: (i) observation (ii) medical intervention (iii) surgery (iv) radical eradication and reconstruction [42]. The embracement of bisphosphonates as a treatment resulted from a better understanding of the etiology of this illness. It inhibits osteoclast activity by binding to bone surfaces, particularly those undergoing active resorption, acting as a biochemical barrier to bone resorption [43]. Posnick and Costello recommended lifelong continuous follow-up for FD [10, 44]. Recurrence of FD is uncommon in adults but more prevalent during adolescence [45].

## Conclusion

FD can present clinically in varied forms which may appear as the collision of different pathologic processes. Currently, treatment for FD is limited, but with a greater understanding of the pathophysiology, we may be able to effectively manage these individuals. Even though the prognosis for FD is favourable, a risk of malignant transformation exists. The likelihood of a recurrence or continued growth of the lesion necessitates a long-term follow-up. With advances in molecular pathology, we now have a better knowledge of the pathophysiology of FD. This review outlines present knowledge on FD, emphasizing the need to understand its molecular pathogenesis alongside its clinical, radiographic, and histological characteristics, as well as essential aspects relevant to differential diagnosis and patient management.

## Data Availability

All data generated or analysed during this study are included in this published article.

## Abbreviations

FD: Fibrous dysplasia
ALP: Serum total bone alkaline phosphatase
U/L: Units per liter
WNT: Wingless-related integration site
GNAS1: Guanine Nucleotide binding protein, alpha stimulating
BFOLs: Benign fibro-osseous lesions
AMP: Cyclic adenosine monophosphate
BMSC: Bone marrow stromal cells
IL–6: Interleukin–6
OHP: Urine hydroxyproline
OC: Osteocalcin
D–Pyr: Urine deoxypyridinoline
CTX: C-telopeptide of type I collagen breakdown products
BSP: Bone sialoprotein
FGF23: Fibroblast growth factor–23
CT: Computed tomography
MRI: Magnetic resonance imaging
CD117: Cluster of Differentiation 117
OPG: Orthopantomogram
